# Change and Tracking of Physical Fitness Among Children Aged 5–12 Years: A Systematic Review

**DOI:** 10.3390/sports14030110

**Published:** 2026-03-11

**Authors:** Priscyla Praxedes Gomes, Carla Santos, José Maia, Peter T. Katzmarzyk, Sara Pereira

**Affiliations:** 1 Centre of Research, Education, Innovation and Intervention in Sport (CIFI2D), Faculty of Sport, University of Porto, 4200-450 Porto, Portugal; priscylapraxedes@hotmail.com (P.P.G.); carlass@fade.up.pt (C.S.); sarasp@fade.up.pt (S.P.); 2 Research Center in Sport, Physical Education, and Exercise and Health (CIDEFES), Faculty of Physical Education and Sports, Lusofona University, 1749-024 Lisboa, Portugal; 3 Pennington Biomedical Research Center, Baton Rouge, LA 70808, USA; peter.katzmarzyk@pbrc.edu

**Keywords:** physical fitness, longitudinal, children 5 to 12 years, changes, tracking

## Abstract

**Background:** Understanding physical fitness (PF) trajectories during childhood is essential because they reflect developmental differences and indicate whether early fitness levels predict later outcomes. Clarifying PF tracking is important for school monitoring, early identification of at-risk children, and planning targeted interventions. **Objective:** To synthesize evidence on change of PF among children aged 5–12 years and, secondarily, to descriptively analyze stability (tracking) of PF components. **Methods:** Searches were conducted in PubMed, Scopus, PsycINFO, and Web of Science covering the last decade (to May 2025). Longitudinal studies assessing at least one PF component in children aged 5–12 years were included. Data extraction included study/sample characteristics, PF components, assessment tools, statistical methods, and outcomes. Methodological quality was assessed with the NIH Quality Assessment Tool for Observational Cohort Studies. **Results:** From 33,995 records, 18 studies met the criteria, with sample sizes from 147 to 1148 children and follow-up from 12 to 48 months. Most studies reported improvements in aerobic, musculoskeletal, and motor fitness, while flexibility showed mixed results. Boys generally outperformed girls in aerobic, motor, and musculoskeletal fitness, whereas girls performed better in flexibility. Stability coefficients, analyzed in a set of four studies, varied across PF components, and results should be interpreted with caution. **Conclusions:** PF generally improves during childhood, with sex-specific patterns and low-to-moderate stability, particularly for motor fitness.

## 1. Introduction

Physical fitness (PF) is a complex and multifactorial construct encompassing the human body’s ability to perform movements and carry out daily tasks that require physical effort [1]. PF comprises several components, including aerobic, motor, and musculoskeletal fitness, and is considered a marker of health, underlying an active and functional lifestyle [1,2,3] during childhood, adolescence, and adulthood.

It has been reported that appropriate levels of PF during childhood are linked to better metabolic health indicators [4,5], greater bone mass and density [6], improved quality of life [7], and higher cognitive performance [8]. Further, evidence suggests that these benefits tend to persist throughout different stages of life [9]. On the other hand, low levels of PF are associated with cardiometabolic risk factors and lower quality of life. For example, a study of children aged 7 to 10 years found that those with a combination of overweight and low aerobic fitness had a higher prevalence of lipid (HDL-c, triglycerides) alterations and greater odds of having three or more cardiometabolic risk factors, compared to those with normal weight and adequate fitness [10]. In addition, Rodrigues de Lima et al. [11] showed that lower musculoskeletal or aerobic fitness in children and adolescents was associated with obesity, high blood pressure, insulin resistance, and unfavorable body composition. For these reasons, it is plausible that reduced PF in childhood compromises both immediate cardiometabolic health and increases the risk of disease in adulthood, reinforcing the importance of early interventions to promote physical activity and improve PF at the school level. Consequently, monitoring PF from early childhood is essential, as this is an important window of opportunity for the development of PF.

Moreover, the increased use of screen-based technologies and digital media has contributed to higher sedentary behavior and reduced spontaneous physical activity [12]. Globally, gaps between physical education policies and implementation, limited time, and quality concerns restrict effective physical education and school-based physical activity [13]. Environmental and socioeconomic disparities, including limited access to safe outdoor spaces, sports facilities, and community programs, further constrain opportunities for physical activity [14,15,16]. The COVID-19 pandemic and associated restrictions exacerbated these trends, resulting in prolonged reduced physical activity, disrupted routines [17], and declining PF levels among pediatric populations [18]. Finally, socioeconomic status, school-related factors (such as access to physical education, facilities, and extracurricular activities), and cultural differences between countries may substantially influence children’s PF levels and developmental trajectories, contributing to variability in longitudinal findings [19,20]. Therefore, monitoring PF from early childhood is essential, as this is an important window of opportunity for the development of PF components and their health effects.

Developmental changes in PF refer to absolute or relative variation in children’s trajectories over time [21]. This variation may include increases, decreases, or no change in performance across PF components [22,23]. These trends may reflect the impact of biological, behavioral, and environmental factors on children’s motor development [24]. Furthermore, there is significant interest in studying PF tracking, as it helps understand the stability of health-related traits over time and identify sensitive periods for intervention [9,25]. Tracking refers to the tendency of individuals to maintain their relative position within a group over time [21,26]. Although statistical approaches to evaluating tracking can be complex [27,28], it is often estimated from autocorrelations (r) across ages. For example, PF data from a longitudinal study conducted by True et al. [29] on American children of both sexes over 12 years showed that tracking was low-to-high (boys: 0.21 ≤ r ≤ 0.79; girls: 0.23 ≤ r ≤ 0.89). Other studies indicate that this trend can extend into adulthood [30,31]. However, systematic reviews that summarize change and stability in PF are important for the reasons discussed above, as studies often differ in their testing methods, follow-up periods, assessment approaches, and statistical analyses. These methodological differences across studies lead to heterogeneity and inconsistent results and conclusions.

Considering the importance of PF components as potential protective factors from early childhood [11,32], their relevance for long-term health outcomes [33], and the recognition of childhood as a sensitive window of opportunity for the development of these components [5], synthesizing the available longitudinal evidence is essential to understand how PF evolves during this time period. However, despite earlier findings reporting low-to-moderate tracking coefficients [9,25], the following questions remain unclear: How do PF levels change and track over time among children aged 5–12 years, and do these trajectories vary by sex? Therefore, based on available evidence from the last decade, the primary aim of the present study was to analyze longitudinal changes in PF levels among children aged 5–12 years. A secondary aim was to provide a descriptive overview of PF stability (tracking).

## 2. Materials and Methods

### 2.1. Protocol Registration

The protocol for this review was registered in PROSPERO (CRD420251042797). Moreover, the systematic review followed the PRISMA statement for reporting systematic reviews [34]. The PRISMA checklist is available in Supplementary File S1.

### 2.2. Study Inclusion Criteria

This systematic review considered data only from longitudinal studies (pure, mixed, or prospective) that monitored changes and/or stability (tracking) in children’s PF components aged 5 to 12 years. The studies included had to meet the following criteria: (1) published in English, Portuguese, French, or Spanish; (2) conducted with children aged 5 to 12 years; (3) longitudinal analysis of at least one PF component; (4) aiming to analyze change and stability, or tracking, in PF component(s); (5) quantitatively presented their results, and (6) published within the last 10 years. The decision to limit the search to the last decade was twofold. First, it ensures the inclusion of studies employing current methodological standards and up-to-date assessment batteries, consistent with criteria established in previous systematic reviews [35,36,37,38]. Second, it allows for a synthesis of evidence that reflects contemporary trends in PF, which have been significantly influenced by recent shifts in lifestyle behaviors, such as the rise in screen-based technologies, distinct from earlier decades [39]. Review articles, validation studies, conference abstracts, monographs, dissertations, theses, commentaries, brief reports, and studies conducted with special populations, namely children with disabilities, developmental delays, or metabolic disorders (e.g., hypertension, diabetes, thyroid disease) were excluded.

### 2.3. Search Methods for Study Identification

This systematic review was conducted in two phases. Phase 1 involved defining the objective and the search protocol (search terms, search strategy, databases for inclusion, and inclusion and exclusion criteria). The descriptors and terms were searched using MeSH (Medical Subject Headings). The search was conducted in PubMed, Scopus, PsycINFO, and Web of Science on 5 May 2025. The structured search used descriptors combined with Boolean operators to identify eligible studies. The strategy included terms related to the target population (Child OR “school age” OR “school-age” OR “primary school”), the related PF components (“physical fitness” OR strength OR motor OR “motor fitness” OR “cardiorespiratory fitness” OR flexibility OR muscular OR “muscular fitness” OR “musculoskeletal fitness” OR “muscle strength” OR “muscular endurance” OR agility OR speed OR “physical performance” OR “health-related fitness”) and the study design (tracking OR stability OR longitudinal OR cohort OR “prospective studies”). The search was applied to the title and abstract fields of the selected databases (see Supplementary File S2 for more details). No manual search of the studies’ reference lists was conducted; the selection was based exclusively on the databases searched.

In phase 2, two independent co-authors (PP and CS) evaluated the titles and abstracts of potentially relevant articles. Subsequently, both researchers reviewed a full copy of the articles that met the initial criteria. The review was carried out using Rayyan software version 1.7.1 (http://rayyan.qcri.org), a web- and mobile-based platform for systematic reviews [40]. Screening and identifying studies for inclusion in the review were conducted at this stage. Titles and/or abstracts of studies retrieved using the search strategy and those from additional sources were selected independently by PP and CS to identify studies that potentially met the inclusion criteria described above. The full text of these potentially eligible studies was independently reviewed and evaluated for eligibility by PP and CS. Any disagreement between them regarding the eligibility of specific studies was resolved through discussion with a third co-author (SP). This process minimized selection bias and ensured consistency in study inclusion. A standardized, pre-piloted form was used to extract data from included studies to assess quality and synthesize the data. Discrepancies were resolved through discussion, and if the two initial co-authors could not reach a consensus, the third co-author was consulted.

### 2.4. Data Extraction

A standardized data extraction form was developed and piloted. Extracted data included study characteristics (e.g., author, year, country), sample characteristics (e.g., age, sex, sample size), PF components assessed, time intervals between assessments, statistical methods used to assess change (e.g., repeated measures ANOVA and/or mixed models) and stability (e.g., Pearson/Spearman auto-correlations, intraclass correlation, kappa coefficient), as well as the main outcomes. Data were coded and organized using Excel and reference management software.

### 2.5. Quality Assessment

The quality of the studies was assessed using the U.S. National Institutes of Health (NIH) questionnaire [41], which evaluates essential aspects of the research, such as clarity in defining the population, measurement validity, and bias control [42]. The questionnaire uses a method to analyze study quality by classifying items as “Yes,” “No,” or “Not reported.” After completing this quality assessment of the studies, a database of results was created. The assessment comprises 14 questions covering topics ranging from defining the research question to controlling for confounding variables. The complete risk of bias assessment was organized according to three steps: (1) two co-authors independently answered the 14 questions for the same papers; (2) a third co-author compiled an overview of the assessments; (3) the third co-author resolved any differences to ensure consistency in assessing the remaining articles.

## 3. Results

### 3.1. Study Screening Process

**Figure 1:**
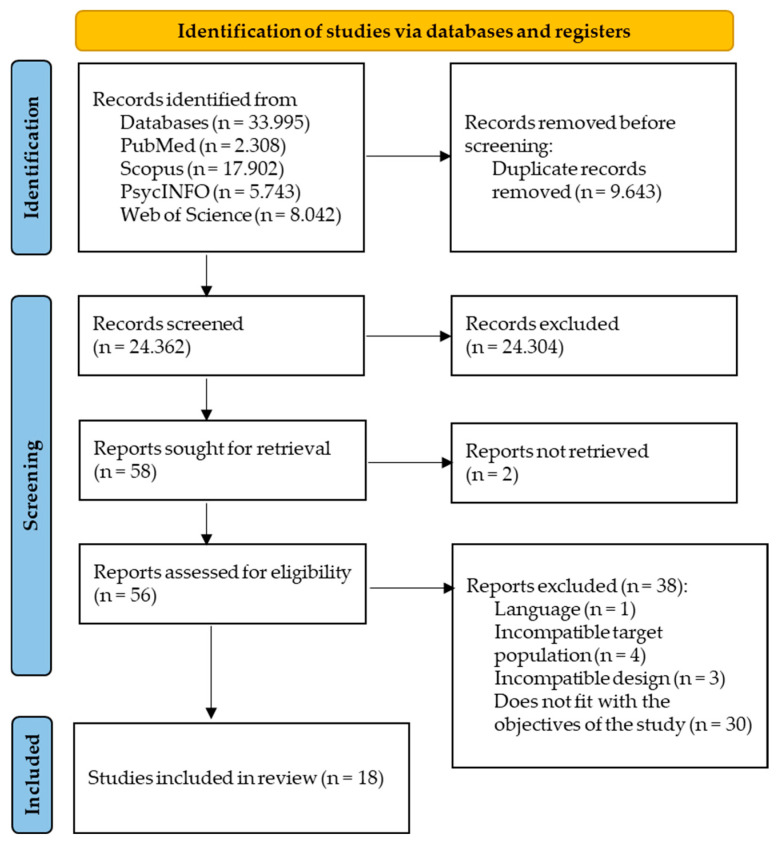
PRISMA diagram of included studies.

Figure 1 shows the PRISMA diagram. The initial search identified 33,995 potential articles from PubMed, Scopus, PsycINFO, and Web of Science databases. A total of 24,362 records were identified after duplicates were removed. After screening titles and abstracts, 58 full-text articles were assessed for eligibility. Two records were not retrieved because they were not indexed in the databases searched at the time of data collection. A total of 56 studies were assessed for eligibility. Of these, one did not meet the language criterion (written in German), four did not meet the established age criterion, three were cross-sectional studies, and thirty did not meet the expected outcome, i.e., PF components of the present review. After this assessment, 18 studies remained eligible for the present systematic review [43,44,45,46,47,48,49,50,51,52,53,54,55,56,57,58,59,60].

### 3.2. Study Selection

#### 3.2.1. Characteristics of Included Studies

Studies were carried out with children of different nationalities, including Austrian [44,47,48,49,54,55,56,57], Brazilian [59,60], Chinese [45], German [45,53], Estonian [50], Finnish [46], French [58], Portuguese [51,52], and English [43]. The 18 studies retrieved for review included 17 written in English and 1 in French. Study samples varied considerably from 147 (Estonia) to 1148 (Finland). Children’s ages ranged from 5 to 12 years, with initial averages of 6.3 ± 0.5 to 11.3 ± 0.3 years. The studies lasted 12 to 48 months, with varying numbers of assessment points ranging from 2 to 8. For included studies that originated from the same cohorts, for example, the Austrian [44,47,48,49] and Brazilian [59,60] cohorts, we adopted the criterion of including more than one publication when they reported different outcomes of interest (e.g., aerobic fitness in one study and musculoskeletal fitness in another). If multiple publications from the same cohort reported the same outcome, we included only the most recent or most relevant study to avoid duplication.

#### 3.2.2. Physical Fitness Measurements

1. Physical Fitness Batteries

Tests were performed using different motor batteries. Seven studies applied tests from the German Motor Test Battery (DMT6-18) [44,45,53,54,55,56,57]. Three studies used the EUROFIT battery [43,51,58], and three studies employed the Austrian AUT FIT battery [47,48,49]. One study used tests from the PREFIT battery [50]; three other studies combined tests from different batteries [51,52,58], including AAHPERD, EUROFIT, Diagnoform, and Fitnessgram. One study applied EUROFIT and AAHPERD tests [51], one used AAHPERD and Fitnessgram [52], and one study used the Diagnoform and EUROFIT batteries [58]. Three studies did not explicitly report the test battery used in their methodology [47,60,61]. Finally, one study used a specific test (the Cooper test, adapted for children) that does not belong to a battery [61].

2. Physical Fitness Tests

The included studies used a variety of tests to assess different components of PF. We relied on Bouchard and Shephard [62] as well as in the IOM report [1] to cluster tests used into the different batteries into the following components: aerobic fitness, musculoskeletal fitness, and motor fitness. Aerobic fitness was measured using the 6 min run in ten studies [44,45,47,48,49,53,54,55,56,57], the 9 min running test used in two studies [59,60], and the PACER in four studies [43,50,58].

Musculoskeletal fitness was assessed via different physical tests. The handgrip test was used in three studies [43,50,51], while medicine ball throwing was used in two studies [47,48]. The standing long jump was the most commonly used test, appearing in 14 studies [43,44,45,47,48,50,51,52,53,54,55,56,57,58]; the push-up test was applied in 8 studies [44,45,46,53,54,55,56,57]; and the sit-up test was used in 9 studies [44,45,46,53,54,55,56,57,59,60].

Flexibility was assessed using the sit-and-reach test in four studies [43,52,53,59], and, in five additional studies, using the stand-and-reach test [44,54,55,56,57].

The motor component included agility and speed tests. Agility was assessed using the 4 × 10 m running test in five studies [47,48,50,51,52]. Speed, in turn, was measured using the 20 m sprint in 7 studies [44,45,53,54,55,56,57] and the 50-yard dash in 1 study. Table 1 provides a detailed description of the characteristics of the 18 studies.

### 3.3. Risk-of-Bias Assessment

The methodological quality of the included studies was assessed using 14 previously defined criteria, which include fundamental aspects of robustness in longitudinal studies (see Supplementary File S3 for more details). All studies (100%) met the requirements regarding clarity of objectives, definition of the target population, validity and reliability of measurements, consistency of measurement procedures over time, adequate statistical analysis, and presentation of results with accuracy indicators (items 1, 2, 7, 9, 10, 11). Eighty percent of the studies reported participation rates, and 94.4% stated sample losses. On the other hand, only 11% of the studies justified the sample size (item 5), and 13% present a follow-up loss rate of less than 20% after the beginning of the study (item 13). None of the included studies blinded the outcome assessor (item 12). It should be noted, however, that blinding of assessors is especially relevant in intervention studies. In the observational studies analyzed here, the absence of this procedure was not considered a significant source of bias. All studies scored between 9 and 13 points.

### 3.4. Outcome

Results were presented as either the average raw scores for each test [43,46,50,51,52,53,60], individual z-scores for each test [44,45,47,48,49,56,57], and an overall PF z-score [54,55,56,58,59].

### 3.5. Main Results

#### 3.5.1. Changes in Physical Fitness Components

Table 2 presents the results regarding changes in children’s PF. In general, the studies showed significant improvements in PF components over time. Thirteen studies found increases in components of aerobic fitness [45,46,47,48,49,50,52,53,54,55,56,59,60], and one study found increases only in the summer months [44]. Thirteen studies found an increase in the performance of the musculoskeletal component [43,44,45,46,47,48,50,51,52,53,54,55], and nine in the motor component [45,47,48,50,51,52,53,54,55]. Seven studies reported that boys outperformed girls in aerobic, musculoskeletal, and motor fitness components [44,45,46,47,48,51,53], while girls excelled in the flexibility component [44]. Moreover, sex differences varied across assessment periods in one study [46]. Four studies found no significant sex differences [43,50,56,57]. Additionally, other studies analyzed changes by sex or adjusted their results for this variable [49,52,55,58,59,60]. Furthermore, a study reported that overweight children have lower PF compared to non-overweight peers [54]. In a comparative study between German and Chinese children, German children outperformed their Chinese peers on some tests [45]. Three studies analyzed changes in PF before, during, and after COVID-19 isolation measures [43,47,48]. One study showed that children who participated in sports clubs performed better in aerobic, musculoskeletal, and motor fitness [48]. Additionally, there are signs of decline in some components of PF over time [43,44,46,47,48,52,58].

#### 3.5.2. Physical Fitness Tracking

A total of 4 studies analyzed PF tracking or one of its components [53,58,59,60], and their detailed descriptions are provided in Supplementary File S4. Aerobic fitness coefficients ranged from 0.357 to 0.625 in boys and from 0.224 to 0.517 in girls. Musculoskeletal fitness values ranged from 0.297 to 0.679 in boys and from 0.235 to 0.624 in girls. Motor fitness coefficients were higher and more consistent, ranging from 0.552 to 0.633 in boys and from 0.340 to 0.550 in girls. Flexibility correlations ranged from 0.512 to 0.593 in boys and from 0.615 to 0.655 in girls. Finally, general PF, assessed using the kappa coefficient, with values of 0.444 for boys and 0.335 for girls.

## 4. Discussion

This systematic review aimed to gather and critically analyze the evidence on changes and tracking in PF levels in children aged 5 to 12 years. By synthesizing longitudinal studies from the past 10 years, this review expands the current evidence base. A total of eighteen studies met the eligibility criteria. Overall, the results suggest a general trend of improvement in most components of PF in children over time within this age range. However, the magnitude of change varied among components, and decreases were also observed. Furthermore, some studies highlighted that individual, environmental, and behavioral factors can influence these changes. These findings underscore the complexity of PF development in children but should be interpreted with caution due to methodological differences across studies. It is essential to note that, to date, no systematic review has thoroughly examined the changes and tracking of the different PF components in children aged 5 to 12 over the past decade.

### 4.1. Changes in Physical Fitness Components

The positive trajectory of musculoskeletal fitness is consistent with previous findings showing that its components tend to increase with age during childhood and adolescence [24]. Physical growth and biological maturation contribute to this process, as increased lean muscle mass improves force production capacity [63]. However, during the prepubertal phase, musculoskeletal fitness gains are predominantly due to neural and coordinative adaptations, whereas hypertrophic gains become more evident after puberty, especially in boys, due to increased testosterone levels [64], which explains sex differences around puberty. This means that these gains may be linked to children’s involvement in physical activity, which may lead to better neuromuscular adaptation. Longitudinal studies that monitor musculoskeletal fitness components are particularly relevant, as they are considered important markers of overall health in childhood. It is positively associated with healthy body composition, bone mineral density, and functional capacity, and is also a predictor of cardiometabolic diseases [3,65]. A systematic review of longitudinal observational studies found that, even in the absence of specific interventions, children show gradual increases in musculoskeletal with age, reflecting the combined effects of physical growth, biological maturation, and participation in daily physical activities [3,65]. Therefore, monitoring musculoskeletal fitness components during the school year is essential to diagnose potential problems early and intervene appropriately.

Improved aerobic fitness results from the interaction between biological, behavioral, and environmental factors [66]. Like musculoskeletal fitness, aerobic capacity also evolves with physical growth, biological maturation, and changes in body composition [67]. Previous studies showed that absolute aerobic capacity (VO_2max_) more than doubles between the ages of 6 and 12 in boys, with girls following a similar trend but at lower absolute levels. However, when expressed relative to body mass, aerobic fitness tends to remain stable or even decline in girls. This progression reflects the maturation of the cardiovascular and respiratory systems, with increases in stroke volume and lung capacity [67]. In addition to biological factors, active behaviors and regular sports practice play a decisive role in this increase [66]. Children who participate in sports activities and maintain a healthy body weight tend to perform better in endurance tests, reinforcing the importance of active habits from childhood [68]. However, the broad age range analyzed (5–12 years) encompasses different developmental stages, contributing to variability in results across studies. Physical growth and biological maturational differences should therefore be considered with caution when interpreting and generalizing the findings.

Positive changes in motor fitness components, such as agility and speed, have already been identified in other studies [69,70]. Such improvements may be associated with increased opportunities for sports practice and the emphasis on short-duration, high-intensity activities in school and recreational settings. However, the magnitude of these improvements is not general, as it is influenced by contextual, socioeconomic, and cultural factors that shape habitual levels of physical activity [71]. The development of agility and speed results from the interaction between neuromuscular adaptations, physical growth, and the refinement of gross motor coordination [24]. Regular practice of games and sports intensifies this progression, making movements more efficient and controlled. The development of these capacities, however, is not a linear process. Studies show that improvements in agility and speed fluctuate during growth, especially during periods of rapid physical development [64]. A recent review confirms that motor and musculoskeletal fitness are associated with bone mineral density in children, reinforcing their role in childhood bone health [72]. Therefore, continuous monitoring of motor fitness is essential to promote balanced physical development.

The variability observed in the development of flexibility suggests that this component is particularly influenced by sex-specific maturation patterns and sociocultural factors that shape children’s exposure to activities requiring a wide range of motion. Girls consistently outperformed boys [73]. These gender differences are often attributed to behavioral and sociocultural factors [74]. From childhood, girls are often encouraged to participate in activities that require a greater range of motion, such as dance, gymnastics, and ballet. At the same time, boys are directed toward disciplines that emphasize musculoskeletal and motor fitness [75]. This differentiation in stimuli reflects cultural norms about what is appropriate for each sex. Moreover, flexibility is joint-specific and influenced by anatomical and maturational factors. Girls generally maintain a greater range of motion due to earlier maturation and differences in pelvic structure, whereas boys may experience transient declines in flexibility during periods of rapid growth [24]. Such evidence highlights the importance of promoting equitable opportunities for the development of flexibility between boys and girls.

Despite the predominance of positive results, some studies reported declines in PF components, particularly aerobic fitness. Reductions in musculoskeletal fitness and flexibility have also been observed, although less frequently [43,58,60]. These negative changes have been attributed to behavioral, contextual, and biological factors. Studies spanning the COVID-19 pandemic have identified marked reductions in aerobic fitness, musculoskeletal fitness, and flexibility resulting from the suspension of school and sports activities, as well as increased sedentary behavior [43,47,48,49]. Furthermore, differences in biological maturation, socioeconomic status, and access to physical activity recreational spaces may contribute to temporary stagnation or regression in some fitness indicators [46,51,56]. These results underscore the importance of considering the social, environmental, and individual contexts when examining children’s PF development. It should be noted that some studies used adjustment variables such as sex, age, school year, body mass index, weight, and stature [43,44,45,46,54,55,56,57]. Contextual aspects, such as school location (urban or rural) and nationality, were also considered by some studies [45,48,55]. Lifestyle factors, such as participation in sports clubs, outdoor playtime, and moderate physical activity [47,48,49,51,56]. Two studies also highlighted the influence of parental educational level and family physical activity [51,56], reinforcing the role of family context in children’s physical development. Childhood PF changes are influenced by socioeconomic status, which affects access to sports, recreational spaces, and physical activity opportunities, thereby shaping PF development. Consequently, behavioral factors, including sports participation, physical activity, and sedentary behaviors, significantly influence PF trajectories.

### 4.2. Tracking of Physical Fitness Components

The second objective of this review was to identify the stability of PF in children aged 5 to 12 years. It is important to note that only four studies [49,54,55,56] specifically analyzed tracking coefficients. Therefore, the following synthesis should be interpreted as a descriptive overview of these specific cohorts rather than a generalized trend. Results indicate that PF tracking showed moderate levels of stability over time, with variations between components and between sexes. Regarding musculoskeletal fitness, studies presented coefficients of 0.297 ≤ r ≤ 0.679 in boys and 0.235 ≤ r ≤ 0.624 in girls, suggesting a tendency toward slightly higher tracking values among boys. This tendency may be related to early sex differences in neuromuscular development and habitual physical activity patterns [70]. Even before puberty, boys tend to have greater relative lean mass and better intermuscular coordination, factors that favor more consistent gains in musculoskeletal fitness and stability over time [31,76,77]. Furthermore, longitudinal studies indicate that boys participate more frequently in vigorous and endurance activities, while girls tend to engage more in low-intensity activities, which may contribute to greater stability of musculoskeletal fitness in boys [78].

For aerobic fitness, the correlation coefficients reported by the studies were 0.357 ≤ r ≤ 0.625 in boys and 0.224 ≤ r ≤ 0.517 in girls, with boys having higher coefficients compared to girls. The higher stability observed in boys is consistent with their generally greater aerobic capacity, which is associated with larger cardiac dimensions and higher hemoglobin levels [67,76]. Conversely, greater variability in girls’ activity patterns and maturational timing may contribute to lower stability in aerobic indicators and more consistent participation in moderate-to-vigorous activities [31,78]. Thus, differences in habitual motor behavior and physiological determinants partially explain the higher correlation coefficients for aerobic fitness observed in boys compared to girls during childhood. The higher tracking coefficients observed for agility and speed in boys can be explained by biological and behavioral factors. Studies suggest that, even in childhood, boys have greater relative muscle mass and muscle power in their lower limbs, factors that favor the consistent execution of rapid movements and changes of direction [31]. Furthermore, boys tend to participate more frequently in dynamic physical activities, such as team sports, which promote the development of speed and agility, reinforcing the stability of these abilities over time. Thus, combined biological and behavioral differences explain the greater stability of agility and speed in boys. Regarding the flexibility component, studies have shown a trend toward high stability over time, particularly in girls. In boys, correlation coefficients ranged from 0.512 to 0.593, while in girls, they ranged from 0.615 to 0.655. This advantage is attributed to both anatomical and behavioral factors, as girls typically exhibit earlier maturational gains in joint mobility and are more often involved in activities such as dance and gymnastics [79,80]. Tracking differences across PF components reflects biological and behavioral mechanisms. Motor components (agility, speed) show greater stability in childhood due to early neuromuscular coordination, which remains stable once established. Aerobic fitness and flexibility fluctuate more, influenced by physical activity, body composition, and environmental factors, which explains the variability in tracking coefficients across studies.

### 4.3. Methodological Implications

The studies included in this review showed significant heterogeneity in several methodological aspects. A total of 61% of the studies were conducted in European countries, including France, Finland, Portugal, Estonia, Germany, and Austria, reflecting the well-established tradition of research on health-related PF and the presence of national school monitoring programs [44,45,46,47,48,49,53,54,55,56,57]. The duration of the studies and the number of assessment points varied from 12 to 48 months and from 2 to 8 assessments, respectively, which may influence the sensitivity for detecting changes and the interpretation of PF component stability [24,81]. Regarding the test batteries, a wide diversity of protocols was observed, including individually validated tests such as the adapted Cooper test, reflecting the multidimensional nature of PF, but making comparisons between studies difficult. The studies used varied statistical approaches, from repeated-measures ANOVA to multilevel models and correlations, to more robust methods such as autoregressive models with latent variables, which offer greater sensitivity to capture changes and stability and minimize methodological biases [81,82]. As for methodological quality, the studies presented high overall quality, meeting criteria of clarity of objectives, definition of the target population, validity and reliability of measurements, and consistency of procedures. Only 11% justified the sample size, and 13% reported an attrition rate of less than 20%, highlighting a potential risk of bias due to participant loss and limitations in interpreting developmental trajectories. Therefore, the results should be interpreted with caution, as some conclusions about the development of PF may be underestimated or overestimated due to insufficient samples or losses over time [83]. Importantly, this methodological heterogeneity has direct implications for the estimation and comparability of tracking coefficients across studies. Differences in test protocols, scoring systems, and measurement precision across batteries may lead to variability in stability estimates, particularly when tracking is assessed using autocorrelations over differing follow-up intervals. Some PF components, such as aerobic fitness and flexibility, may be more influenced by test-specific characteristics and protocol design, whereas motor components (e.g., agility and speed) tend to show greater consistency across testing approaches. Consequently, comparisons of tracking coefficients across studies using different PF test batteries (e.g., DMT6–18, EUROFIT, AUT FIT) should be interpreted with caution, as these instruments assess related but not identical markers of PF. This methodological diversity primarily affects the precision and interpretation of stability estimates rather than the underlying developmental trajectories of PF.

### 4.4. Practical Implications and Future Directions

The findings of this review have important practical implications for promoting PF in children. Given that PF development is influenced by biological, behavioral, and environmental factors, it is essential to regularly monitor components such as musculoskeletal fitness, aerobic fitness, flexibility, and motor skills to identify potential deficiencies early and intervene appropriately. Promoting regular physical activity and sports, while reducing sedentary behavior, should be encouraged in both school and recreational settings, ensuring equitable opportunities for boys and girls. Continuous, structured programs throughout the school years can significantly contribute to children’s overall physical fitness, supporting healthy habits that extend into adulthood.

Understanding these trajectories is essential for designing more effective interventions, school programs, and health policies that promote healthy PF in childhood and help prevent long-term issues, namely sedentary lifestyles, obesity, and related chronic diseases. In this context, the heterogeneity observed across studies underscores the need for future longitudinal research to more systematically account for physical growth, as well as socioeconomic and behavioral factors, and to apply more appropriate statistical techniques that accurately capture both the changes and the stability of PF in school-aged children within their varied living contexts.

### 4.5. Limitations of the Current Review

Despite adopting broad, well-structured search strategies, it is not always possible to identify all relevant studies, particularly unpublished ones. Furthermore, the selection of databases, keywords, and languages may have restricted the scope of the research. Language bias is also a limitation, as the predominant inclusion of studies published in English may have resulted in the exclusion of relevant evidence in languages other than English. Another aspect to consider is the heterogeneity among the studies analyzed, which presents differences in populations, methods, interventions, and outcome measures, making comparison and synthesis of results difficult. Moreover, although methodological rigor was maintained, the process of selecting and evaluating studies involves interpretation by the reviewers, which may introduce human bias into the analysis. It is important to acknowledge that a substantial proportion of the included studies originated from Austria and Brazil, and this issue must be clearly understood when deriving putative generalizing trends. The synthesis of results should be interpreted with caution, given the specific environmental and educational contexts of these populations, limiting the generalization of the trends to a global scale. Therefore, restricting studies to those published in the last 10 years, while ensuring methodological currency, may have excluded older longitudinal cohorts. This limitation is particularly relevant for tracking. Earlier studies conducted in less sedentary environments might show different stability patterns. Therefore, the conclusions regarding tracking presented here should be interpreted specifically within the context of the last decade. Finally, the synthesis of PF changes was limited to a binary description of directionality (increase vs. decrease) because standardized effect sizes were not available across studies. This approach simplifies complex developmental trajectories and does not account for the magnitude or clinical relevance of the changes. Consequently, statistically significant improvements should be interpreted with caution, as they do not reflect the effect size of the observed development.

## 5. Conclusions

This review shows that PF components in children generally improve from 5 to 12 years of age, although the magnitude of change varies across components and between boys and girls. Although data from studies analyzing stability suggest that children tend to maintain their relative fitness rankings over time, and these results should not be generalized, they underscore the importance of early interventions. It is also important to stress that PF development during childhood is influenced by physical growth, physical activity levels, sedentary behaviors, and contextual factors (school, family socioeconomic status and support, and sports participation). From a practical perspective, promoting regular physical activity through school programs, specifically by offering an adequate number of physical education classes and implementing public health policies, is essential to support healthy development and prevent long-term adverse outcomes.

## Figures and Tables

**Table 1:** Studies that analyzed the change and stability of physical fitness in children aged 5–12 years.

Authors	Country	Sample Characteristics			Timeframe		PF Assessment		
		Sample (n)	Gender	Age at Baseline in Years (Mean ± DP)	Duration of Study (Months)	Assessment Points	PF Batteries	Tests	Fitness Metric
Basterfield et al. (2022) [43]	United Kingdom	n = 178	83 girls; 95 boys	9.1 ± 0.6	12	2	EUROFIT	PACER Handgrip strength Standing long jump Sit-and-reach	Average raw scores for each test
Drenowatz et al. (2021) [44]	Austria	n = 214	102 girls; 112 boys	6.9 ± 0.4	48	8	DMT6-18	6 min run 20 m sprint Standing long jump Push-ups Sit-ups Stand-and-reach	Individual z-scores
Hohmann et al. (2021) [45]	China and Germany	n = 577	China (145 boys; 151 girls) Germany (121 boys; 160 girls)	7.8 ± 0.4	12	2	DMT6-18	6 min run 20 m sprint Standing long jump Push-ups Sit-ups	Individual z-scores
Jaakkola et al. (2021) [46]	Finland	n = 1148	583 girls; 565 boys	11.3 ± 0.3	24	3	NR	PACER Curl-up Push-ups	Average raw scores for each test
Jarnig et al. (2022) [47]	Austria	n = 303	147 girls; 156 boys	7.7 ± 0.4	24	3	AUT FIT	6 min run 4 × 10 m shuttle run Standing long jump Medicine ball throw Sit-and-reach	Individual z-scores
Jarnig et al. (2022) [48]	Austria	n = 706	350 girls; 356 boys	8.3 ± 0.7	30	5	AUT FIT	6 min run 4 × 10 m shuttle run Standing long jump Medicine ball throw	Individual z-scores
Jarnig et al. (2024) [49]	Austria	n = 331	159 girls; 172 boys	7.7 ± 0.4	33	3	AUT FIT	6 min run	Individual z-scores
Reisberg et al. (2020) [50]	Estonia	n = 147	72 girls; 75 boys	6.6 ± 0.51	12	2	PREFIT	PACER 4 × 10 m shuttle run Handgrip strength Standing long jump	Average raw scores for each test
Reyes et al. (2023) [51]	Portugal	n = 348	177 girls; 171 boys	5 to 11 y	36	3	EUROFIT, AAHPERD	4 × 10 m shuttle run Handgrip strength Standing long jump	Average raw scores for each test
Rodrigues et al. (2016) [52]	Portugal	n = 472	233 girls; 239 boys	6.3 ± 0.7	48	4	AAHPERD, Fitnessgram	PACER 4 × 10 m shuttle run 50 yards Standing long jump Sit-ups Flexed arm hang Sit-and-reach	Average raw scores for each test
Roth et al. (2018) [53]	Germany	n = 252	Cohort 1: 116 girls, 137 boys Cohort 2: 166 girls, 149 boys	6.9 ± 0.4 boys; 6.8 ± 0.5 girls	48 (Cohort 1) and 36 (Cohort 2)	4 and 3	DMT6-18	6 min run 20 m sprint Standing long jump Push-ups Sit-ups Sit-and-reach	Average raw scores for each test
Ruedl et al. (2018) [54]	Austria	n = 266	120 girls; 146 boys	6.4 ± 0.5	29	5	DMT6-18	6 min run 20 m sprint Standing long jump Push-ups Sit-ups Stand-and-reach	Overall PF z-score and average raw scores for each test
Ruedl et al. (2019) [55]	Austria	n = 265	120 girls; 146 boys	6.4 ± 0.5	36	3	DMT6-18	6 min run 20 m sprint, Standing long jump Push-ups Sit-ups Stand-and-reach	Overall PF z-score
Ruedl et al. (2022) [56]	Austria	n = 265	119 girls; 146 boys	6.9 ± 0.5	48	4	DMT6-18	6 min run 20 m sprint Standing long jump Push-ups Sit-ups Stand-and-reach	Overall PF z-score and Individual z-scores
Ruedl et al. (2024) [57]	Austria	n = 263	118 girls; 145 boys	6.9 ± 0.5	48	8	DMT6-18	6 min run 20 m sprint Standing long jump Push-ups Sit-ups Stand-and-reach	Individual z-scores
Vanhelst et al. (2020) [58]	France	n = 516	262 girls; 254 boys	7.7 ± 0.4	36	2	Diagnoform, EUROFIT	PACER 5 s sprint Standing long jump	Overall PF z-score
Werneck et al. (2019) [59]	Brazil	n = 372	176 girls; 196 boys	9.9 ± 1.1 girls; 8.9 ± 1.1 boys	36	3	NR	9 min run/walk Sit-and-reach Sit-ups	Overall PF z-score
Werneck et al. (2019) [60]	Brazil	n = 372	176 girls; 196 boys	9.0 ± 1.1 girls; 8.9 ± 1.1 boys	36	3	NR	9 min run/walk	Average raw scores for each test

NR—not reported; min = minute; s = seconds; m = meters; AUT FIT = Austrian fitness monitoring tools for schoolkids; AAHPERD = American Alliance for Health, Physical Education, Recreation and Dance; EUROFIT = European Physical Fitness Test Battery; DMT6-18 = Deutscher Motorik-Test 6-18; FitnessGram^®^: The Cooper Institute’s Health-Related Fitness Assessment; PREFIT = Physical Fitness Battery in Preschool Children; Individual z-scores—scores for each component; Overall PF z-score—average of the z-scores obtained from the different components of physical fitness. Assessment points = number of measurement waves; Duration of study (months) = refers to the total monitoring period.

**Table 2:** Change and stability of PF in children aged 5 to 12 years.

Reference	Country	Objective(s)	Independent Variables/ Control Variables/Factors Included in the Analysis	Data Analysis	Change of PF
Basterfield et al. (2022) [43]	United Kingdom	To assess changes in children’s PF, BMI, and HRQoL over 12 months in children aged 8–10, encompassing the first 2020 UK COVID-19 lockdown and school closure period; (2) To explore the associations between the variables.	Baseline BMI z-score, sex, and baseline age	Multiple linear regression, ANOVA	**One year of follow-up** **Increase:** Standing long jump, handgrip strength (right), handgrip strength (left). **Decrease:** PACER, sit-and-reach. * No differences between the sexes.
Drenowatz et al. (2021) [44]	Austria	To examine PF components, change in elementary school children over four years, during the school year and the summer months, in children who were 6 years of age at the beginning of the study.	Sex, age, summer months and School-Year	ANOVA RM	**Four years of follow-up** **Increase:** School-Year: Sit-ups, standing long jump, stand-and-reach. Summer Months: 6 min run, push-ups, sit-ups, stand-and-reach (Improves only in girls over the years). **Decrease:** School-Year: 6 min run, push-ups. * Stand-and-reach: better performance and change in girls; long jump: better performance and change in boys.
Hohmann et al. (2021) [45]	China/Germany	To investigate the relationship between moderate-to-vigorous physical activity, PF, and motor competence in German and Chinese children aged 8–9.	Sex, age, time, nationality	General linear model	**One year of follow-up** **Increase:** 6 min run (superior performance by boys and German children. 20 m run (no significant differences between genders. Standing long jump: better performance by boys and Chinese children. Push-ups: superior performance by German children. Sit-ups: Boys and German children perform better.
					Furthermore, a significant improvement in the performance of Chinese children over time was observed in physical tests.
Jaakkola et al. (2021) [46]	Finland	To identify latent physical performance profiles in children’s motor competence, aerobic fitness, and musculoskeletal fitness; (2) explore transition probabilities in performance profiles over 2 years in children who were 11 years of age at the beginning of the study).	Sex, School, School Class	Latent Transition Analysis	**Two years of follow-up** **Increase:** Push-ups (both, girls consistently higher). Sit-ups (slight improvement in boys). Aerobic fitness (both, peak at T1, small decline at T2 but still > T0). **Decrease:** Push-ups (temporary decrease at T1 before increase at T2). Sit-ups (girls stable, boys lower than girls overall).
Jarnig et al. (2022) [47]	Austria	The aim of this study was to investigate the impact of COVID-19 mitigation measures on the health status and physical fitness of children in Austria (aged 7–10).	Sex, age, region (urban/rural), and the effects of time	ANOVA RM	**Two and a half years** **Increase:** T1–T2: Standing long jump (more pronounced in children from rural schools and in the second grade of the school year). T3–T5 6 min run (no differences by sex, school location or grade; standing long jump (more pronounced in children from rural schools and 2nd grade, no significant differences by sex), medicine ball throw (more pronounced in boys, no differences by school location or grade), 4 × 10 m run (no significant differences by sex or grade). **Decrease:** T1–T3: 6 min run. (No differences by sex, school location, or grade); medicine ball throw (more pronounced in boys), with no differences by school location or grade or by school location or grade. T3–T5: Standing long jump (more pronounced in children from rural schools and 3rd grade, no differences by sex.
Jarnig et al. (2022) [48]	Austria	The aim was to assess changes in physical fitness, health-related quality of life (HRQoL), and body mass index (BMI) over 1 year, spanning the UK COVID-19 lockdowns of 2020 in children aged 7–10 years	Sex, Sports club membership, time points	ANOVA RM	**Three years of follow-up** **Increase:** T1–T2 (2019 to 2020): Standing long jump (improvement in all groups). T2–T3 (2020 to 2021): 4 × 10 m shuttle run (improvement in all subgroups). **Decrease:** T1–T2 (2019 to 2020): 6 min run (more pronounced reduction in girls and children without a sports club), 4 × 10 m shuttle run (reduction in all groups). T2–T3 (2020 to 2021): Standing long jump (reduction in all groups observed). * boys are better than girls at sit-and-reach in T2 to T3.
Jarnig et al. (2024) [49]	Austria	To investigate how body mass index and aerobic fitness of Austrian children aged 7–9 years have evolved, from the period before the COVID-19 pandemic (September 2019) to during the strict mitigation measures (until June 2021) and after the relaxation of these measures (June 2022).	Sex, age, school, class	ANOVA RM	**Two years and eight months** **Increase:** 6 min run after restrictions (P2) in boys and in children participating in sports clubs. **Decrease:** 6 min run from baseline (P0, September 2019) during COVID-19 restrictions (P1) in all subgroups. * After restrictions (P2), performance decreased in girls and children not participating in sports clubs.
Reisberg et al. (2020) [50]	Estonia	To investigate the longitudinal associations of physical activity with body composition and PF components at the 12-month follow-up during the transition from kindergarten to primary school (aged 6–8 years).	Age, sex, BMI, height, Socioeconomic status, education, health conditions	Multiple linear regression, Paired t-test	**One year of follow-up** **Increase:** PACER, 4 × 10 m shuttle run, handgrip strength, standing long jump.
Reyes et al. (2023) [51]	Portugal	To model children’s musculoskeletal fitness developmental trajectories and identify individual differences related to the effects of time-invariant and time-variant covariates (5–11 years).	Age, sex, cohort, birth weight, socioeconomic status, BMI, gross motor coordination, and physical activity	Multilevel modeling	**Three years of follow-up** **Increase:** Handgrip strength (better performance in boys, children with higher BMI, and better motor coordination). Standing long jump (better performance in boys and children with lower BMI, more minutes of MVPA, and better motor coordination), 4 × 10 m shuttle run (better performance was observed in boys and children with better motor coordination, and children with low birth weight and more minutes of MVPA showed lower performance). Handgrip strength increased with age.
Rodrigues et al. (2016) [52]	Portugal	To examine how different developmental pathways of health-related physical fitness and motor competence are associated with overweight and obesity status in children aged 6–10 years.	Sex, BMI, and age	Multilevel modeling	**Four years of follow-up** **Increase:** High Rate of Change (above the 75th percentile): Sit-ups, flexed arm hang, sit-and-reach, PACER, Standing Long Jump, 4 × 10 m shuttle run, and 50 m dash. Average Rate of Change (between the 25th and 75th percentile): Sit-ups, PACER, Standing Long Jump, 4 × 10 m shuttle run, 50 m Dash. Low Rate of Change (below the 25th percentile)—10 m Shuttle Run and 50 m Dash. **Decrease:** Low Rate of Change (below the 25th percentile): Sit-ups, Flexed Arm Hang, Sit-and-reach, PACER, and Standing Long Jump.
Roth et al. (2018) [53]	Germany	The aim of this study was to measure motor development and monitor the physical fitness components of children (6–10 years) in Trier, Germany.	Sex, age, time-by-sex	ANOVA RM, Pearson correlations	**Four and three years of follow-up** **Increase:** LC1 (4-year follow-up)—20 m sprint, standing long jump, sit-ups, push-ups, 6 min run. LC2 (3-year follow-up): 20 m sprint, standing long jump, 6 min run. Significant sex differences were found. Boys generally performed better in the 20 m sprint and 6 min run, while girls showed better results in the sit-and-reach test.
Ruedl et al. (2018) [54]	Austria	To evaluate the development of physical fitness in overweight and non-overweight children (aged 6–10 years) over 2.5 years	Sex, age, time	ANOVA RM	**Two and a half years of follow-up** **Increase:** 6 min run (Significant difference between groups, with an advantage for non-migrants and a time effect, but migrants maintained lower values at all time points). Standing long jump (Significant difference between groups in favor of non-migrants, but no time effect). 20 m sprint, push-ups, and sit-ups: Improvements over time for both groups, initial advantage for non-migrants. Stand-and-reach (Small changes, marginal time effect, and no significant differences between groups).
Ruedl et al. (2019) [55]	Austria	To evaluate the effect of migration background on the development of physical fitness among children from first to third grade (6–10 years).	Sex, age, BMI	ANOVA RM ANCOVA	**Two and a half years of follow-up** **Increase:** 6 min run, standing long jump, 20 m sprint, push-ups, sit-ups, stand-and-reach. * All groups improved over time, with migrant children showing more pronounced gains in some tests, particularly jumping sideward, push-ups, and the 6 min run.
Ruedl et al. (2022) [56]	Austria	To evaluate the impact of modifiable and non-modifiable factors on the development of PF 6–10 years.	Sex, age, BMI, outdoor play, Sports club participation, parental physical activity, TV/computer hours per day	Multiple linear regression analysis, Paired t-test and Wilcoxon signed-rank test	**Four years of follow-up** **Increase:** 6 min sun, 20-metre sprint, stand-and-reach, standing long jump, push-ups, sit-ups. * No gender differences. Greater effects of increased PF were observed in children who were never overweight, played outdoors at least five days a week, participated in sports clubs, and whose parents had a higher level of education and greater physical activity.
Ruedl et al. (2024) [57]	Austria	To compare changes in PF and socioeconomic factors from 6 to 10 years based on different levels of SRH and to examine which changes in the above factors may be associated with higher perceived health in Austrian children.	Sex, age, migratory origin, and BMI	T-test for independent samples, multiple logistic regression analysis	**Four years of follow-up** **Increase:** The group in very good health showed a greater average improvement in overall physical fitness (significant subtests: 20 m run, push-ups, sit-ups, long jump, and 6 min run) compared to the group in good health or below.
Vanhelst et al. (2020) [58]	France	To examine longitudinal changes in PF components among children (aged 7 and 10 years) in northern France and to compare their performance with established French normative values.	Sex, age, and BMI	Wilcoxon signed-rank test	**Three years of follow-up** **Decrease:** 6 min shuttle run, standing long jump, and hopscotch test (all declined in both sexes; the decline in standing long jump was steeper in girls). No significant change was found in the 5 s sprint test.
Werneck et al. (2019) [59]	Brazil	To examine the stability of PF, and the interrelationship among intra-individual changes in fitness and fatness among elementary school children (aged 7–10 years).	Age, biological maturation	Test de Mann–Whitney	**Three years of follow-up** **Increase:** 9 min run/walk distance (with boys performing better), 1 min abdominal test (boys also outperformed girls). **Decrease:** sit-and-reach declined in both sexes.
Werneck et al. (2019) [60]	Brazil	To evaluate aerobic fitness tracking from childhood to adolescence (aged 7–10 years), as well as to test the moderating role of somatic maturation	Sex, height, biological maturation	Logistic regressions with adjusted odds ratio (CI 95%)	**Three years of follow-up** **Increase:** 9 min run/walk performance increased in both sexes, especially boys.

BMI = Body Mass Index; High Rate of Change (above the 75th percentile); PF = Physical Fitness; * = results details; ANOVA RM—Repeated Measures Analysis of Variance; ANCOVA—Analysis of Covariance; MVPA—Moderate-to-Vigorous Physical Activity; CI 95%—95% Confidence Interval; P0 = initial assessment; P1 = second assessment; P2 = third assessment; LC1 = longitudinal cohort 1; LC2 = longitudinal cohort 2. Note: Classification of PF change is based on the statistical significance reported in the original studies. Sample size and follow-up duration are reported in Table 2. The magnitude of change could not be synthesized due to substantial heterogeneity in analytical methods and outcome metrics across studies.

## Data Availability

No new data were created or analyzed in this study. Data supporting the findings of this systematic review are available in the published articles included in the review, all of which are cited in the reference list.

## Supplementary Materials

The following supporting information can be downloaded at: https://www.mdpi.com/article/10.3390/sports14030110/s1, File S1: PRISMA checklist; File S2: Search strategies used in each database. (PROSPERO reference: PROSPERO CRD420251042797); File S3: Methodological analysis and risk of bias of the included studies; File S4: Tracking Physical Fitness in children aged 5 to 12 years.
