# Supplementary material for: Change and Tracking of Physical Fitness Among Children Aged 5–12 Years: A Systematic Review
Source: Sports (Basel). 2026 Mar 11;14(3):110. doi: 10.3390/sports14030110 (PMC13030685; doi:10.3390/sports14030110)
Supplement: Supplementary file 1 [file sports-14-00110-s001.zip › sports-4152890-supplementary.pdf]

Supplementary file S1. PRISMA checklist

PRISMA 2020 Checklist

Section and Topic	Item #	Checklist item	Location where item is reported
**TITLE**			
Title	1	Identify the report as a systematic review.	Page 1
**ABSTRACT**			
Abstract	2	See the PRISMA 2020 for Abstracts checklist.	Page 1
**INTRODUCTION**			
Rationale	3	Describe the rationale for the review in the context of existing knowledge.	Page 2
Objectives	4	Provide an explicit statement of the objective(s) or question(s) the review addresses.	Page 2
**METHODS**			
Eligibility criteria	5	Specify the inclusion and exclusion criteria for the review and how studies were grouped for the syntheses.	Page 3
Information sources	6	Specify all databases, registers, websites, organisations, reference lists and other sources searched or consulted to identify studies. Specify the date when each source was last searched or consulted.	Page 3
Search strategy	7	Present the full search strategies for all databases, registers and websites, including any filters and limits used.	Page 3
Selection process	8	Specify the methods used to decide whether a study met the inclusion criteria of the review, including how many reviewers screened each record and each report retrieved, whether they worked independently, and if applicable, details of automation tools used in the process.	Page 3
Data collection process	9	Specify the methods used to collect data from reports, including how many reviewers collected data from each report, whether they worked independently, any processes for obtaining or confirming data from study investigators, and if applicable, details of automation tools used in the process.	Page 3 and 4
Data items	10a	List and define all outcomes for which data were sought. Specify whether all results that were compatible with each outcome domain in each study were sought (e.g. for all measures, time points, analyses), and if not, the methods used to decide which results to collect.	Page 4
	10b	List and define all other variables for which data were sought (e.g. participant and intervention characteristics, funding sources). Describe any assumptions made about any missing or unclear information.	Page 4
Study risk of bias assessment	11	Specify the methods used to assess risk of bias in the included studies, including details of the tool(s) used, how many reviewers assessed each study and whether they worked independently, and if applicable, details of automation tools used in the process.	Page 3
Effect measures	12	Specify for each outcome the effect measure(s) (e.g. risk ratio, mean difference) used in the synthesis or presentation of results.	Page 3
Synthesis methods	13a	Describe the processes used to decide which studies were eligible for each synthesis (e.g. tabulating the study intervention characteristics and comparing against the planned groups for each synthesis (item #5)).	Page 3
	13b	Describe any methods required to prepare the data for presentation or synthesis, such as handling of missing summary statistics, or data conversions.	NA
	13c	Describe any methods used to tabulate or visually display results of individual studies and syntheses.	Page 4
	13d	Describe any methods used to synthesize results and provide a rationale for the choice(s). If meta-analysis was performed, describe the model(s), method(s) to identify the presence and extent of statistical heterogeneity, and software package(s) used.	Page 4
	13e	Describe any methods used to explore possible causes of heterogeneity among study results (e.g. subgroup analysis, meta-regression).	NA
	13f	Describe any sensitivity analyses conducted to assess robustness of the synthesized results.	NA

Section and Topic	Item #	Checklist item	Location where item is reported
Reporting bias assessment	14	Describe any methods used to assess risk of bias due to missing results in a synthesis (arising from reporting biases).	NA
Certainty assessment	15	Describe any methods used to assess certainty (or confidence) in the body of evidence for an outcome.	NA
**RESULTS**			
Study selection	16a	Describe the results of the search and selection process, from the number of records identified in the search to the number of studies included in the review, ideally using a flow diagram.	Pages 4 and 5
	16b	Cite studies that might appear to meet the inclusion criteria, but which were excluded, and explain why they were excluded.	Page 4
Study characteristics	17	Cite each included study and present its characteristics.	Pages 4 to 9
Risk of bias in studies	18	Present assessments of risk of bias for each included study.	Page 11
Results of individual studies	19	For all outcomes, present, for each study: (a) summary statistics for each group (where appropriate) and (b) an effect estimate and its precision (e.g. confidence/credible interval), ideally using structured tables or plots.	Pages 7 to 10
Results of syntheses	20a	For each synthesis, briefly summarise the characteristics and risk of bias among contributing studies.	Page 12
	20b	Present results of all statistical syntheses conducted. If meta-analysis was done, present for each the summary estimate and its precision (e.g. confidence/credible interval) and measures of statistical heterogeneity. If comparing groups, describe the direction of the effect.	Pages 14 to 19
	20c	Present results of all investigations of possible causes of heterogeneity among study results.	NA
	20d	Present results of all sensitivity analyses conducted to assess the robustness of the synthesized results.	NA
Reporting biases	21	Present assessments of risk of bias due to missing results (arising from reporting biases) for each synthesis assessed.	NA
Certainty of evidence	22	Present assessments of certainty (or confidence) in the body of evidence for each outcome assessed.	NA
**DISCUSSION**			
Discussion	23a	Provide a general interpretation of the results in the context of other evidence.	Page 22
	23b	Discuss any limitations of the evidence included in the review.	Pages 22 to 24
	23c	Discuss any limitations of the review processes used.	Page 27
	23d	Discuss implications of the results for practice, policy, and future research.	Page 27
**OTHER INFORMATION**			
Registration and protocol	24a	Provide registration information for the review, including register name and registration number, or state that the review was not registered.	Page 1
	24b	Indicate where the review protocol can be accessed, or state that a protocol was not prepared.	Page 1
	24c	Describe and explain any amendments to information provided at registration or in the protocol.	NA
Support	25	Describe sources of financial or non-financial support for the review, and the role of the funders or sponsors in the review.	Page 28
Competing interests	26	Declare any competing interests of review authors.	Page 28
Availability of data, code and other materials	27	Report which of the following are publicly available and where they can be found: template data collection forms; data extracted from included studies; data used for all analyses; analytic code; any other materials used in the review.	Pages 28

From: Page MJ, McKenzie JE, Bossuyt PM, Boutron I, Hoffmann TC, Mulrow CD, et al. The PRISMA 2020 statement: an updated guideline for reporting systematic reviews. BMJ 2021;372:n71. doi: 10.1136/bmj.n71.
This work is licensed under CC BY 4.0. To view a copy of this license, visit https://creativecommons.org/licenses/by/4.0/

Supplementary File S2.

Search strategies used in each database. (PROSPERO registre: PROSPERO CRD420251042797)

**Draft search strategy for each electronic database consulted: PubMed, Scopus, PsyINFO, and Web of Science.**<br>For each search listed below, the study time and languages will be applied, and the databases were searched for studies from the last 10 years.

	**Database**	**PubMed**	**Number of articles reached**
	**Descriptors/keywords**		
**1#**	(((((“physical fitness”[Title/Abstract]) OR (strength [Title/Abstract]) OR (motor [Title/Abstract])) OR (“motor fitness”[Title/Abstract])) OR (“cardiorespiratory fitness” [Title/Abstract])) OR (flexibility [Title/Abstract])) OR (muscular [Title/Abstract]) OR (“muscular fitness” [Title/Abstract]) OR (“musculoskeletal fitness” [Title/Abstract]) OR (“muscle Strength” [Title/Abstract]) OR (“muscular endurance” [Title/Abstract])) OR (agility [Title/Abstract]) OR (speed [Title/Abstract]) OR (“physical performance”[Title/Abstract]) OR (“health-related fitness”[Title/Abstract])		1.258.141
**2#**	((((((tracking [Title/Abstract]) OR (stability [Title/Abstract])) OR (longitudinal [Title/Abstract])) OR (cohort [Title/Abstract])) OR (“prospective studies” [Title/Abstract]))		2.082.881
**3#**	(((child [Title/Abstract]) OR (“school age” [Title/Abstract])) OR (“School-Age children” [Title/Abstract])) OR (“primary school” [Title/Abstract])		553.869
**4#**	(#1 AND #2)		125.840
**5#**	(#1 AND #3)		21.157
**6#**	(#2 AND #3)		55.957
**7#**	**((#1) AND (#2)) AND (#3)**		3.315
**8#**	Stipulated time = last 10 years		2.308

	**Database**	**Scopus**	**Number of articles reached**
	**Descriptors/keywords**		
**1#**	TITLE-ABS-KEY (“physical fitness” OR strength OR motor OR “motor fitness" OR "cardiorespiratory fitness" OR flexibility OR muscular OR “muscular fitness” OR “musculoskeletal fitness” OR “muscle strength” OR "muscular endurance" OR agility OR speed OR “physical performance” OR "health-related fitness")		5.957.662
**2#**	TITLE-ABS-KEY (tracking OR stability OR longitudinal OR cohort OR "prospective studies")		6.515.882
**3#**	TITLE-ABS-KEY (child OR “school age" OR “School-Age children” OR "primary school”)		3.964.870
**4#**	**(1# AND 2# AND 3#)**		32.776
	Stipulated time = last 10 years.		20.845

	(LIMIT-TO (EXACTKEYWORD , "Human") OR LIMIT-TO (EXACTKEYWORD , "Child") OR LIMIT-TO (EXACTKEYWORD , "Female") OR LIMIT-TO (EXACTKEYWORD , "Male") OR LIMIT-TO (EXACTKEYWORD , "Article") OR LIMIT-TO (EXACTKEYWORD , "Infant") OR LIMIT-TO (EXACTKEYWORD , "Follow Up") OR LIMIT-TO (EXACTKEYWORD , "Longitudinal Study") OR LIMIT-TO (EXACTKEYWORD , "School Child") OR LIMIT-TO (EXACTKEYWORD , "Motor Performance") OR LIMIT-TO (EXACTKEYWORD , "Longitudinal Studies") OR LIMIT-TO (EXACTKEYWORD , "Child Development") OR LIMIT-TO (EXACTKEYWORD , "Children") OR LIMIT-TO (EXACTKEYWORD , "Follow-Up Studies") OR LIMIT-TO (EXACTKEYWORD , "Motor Skills") OR LIMIT-TO (EXACTKEYWORD , "Motor Development") OR LIMIT-TO (EXACTKEYWORD , "Muscle Strength") OR LIMIT-TO (EXACTKEYWORD , "Childhood") OR LIMIT-TO (EXACTKEYWORD , "Child Health") OR LIMIT-TO (EXACTKEYWORD , "Fitness")) AND (LIMIT-TO (DOCTYPE , "ar"))	
**(1# AND 2# AND 3#)**	*((TITLE-ABS-KEY ("physical fitness") OR TITLE-ABS-KEY (strength) OR TITLE-ABS-KEY (motor) OR TITLE-ABS-KEY ("motor fitness") OR TITLE-ABS-KEY ("cardiorespiratory fitness") OR TITLE-ABS-KEY (flexibility) OR TITLE-ABS-KEY (muscular) OR TITLE-ABS-KEY ("muscular fitness") OR TITLE-ABS-KEY ("musculoskeletal fitness") OR TITLE-ABS-KEY ("muscle strength") OR TITLE-ABS-KEY ("muscular endurance") OR TITLE-ABS-KEY (agility) OR TITLE-ABS-KEY (speed) OR TITLE-ABS-KEY ("physical performance") OR TITLE-ABS-KEY ("health-related fitness"))) AND ((TITLE-ABS-KEY (tracking) OR TITLE-ABS-KEY (stability) OR TITLE-ABS-KEY (longitudinal) OR TITLE-ABS-KEY (cohort) OR TITLE-ABS-KEY ("prospective studies"))) AND ((TITLE-ABS-KEY (child) OR TITLE-ABS-KEY ("school age") OR TITLE-ABS-KEY ("School-Age children") OR TITLE-ABS-KEY ("primary school"))) AND PUBYEAR > 2014 AND PUBYEAR < 2026 AND (LIMIT-TO (EXACTKEYWORD , "Human") OR LIMIT-TO (EXACTKEYWORD , "Child") OR LIMIT-TO (EXACTKEYWORD , "Female") OR LIMIT-TO (EXACTKEYWORD , "Male") OR LIMIT-TO (EXACTKEYWORD , "Article") OR LIMIT-TO (EXACTKEYWORD , "Infant") OR LIMIT-TO (EXACTKEYWORD , "Follow Up") OR LIMIT-TO (EXACTKEYWORD , "Longitudinal Study") OR LIMIT-TO (EXACTKEYWORD , "School Child") OR LIMIT-TO (EXACTKEYWORD , "Motor Performance") OR LIMIT-TO (EXACTKEYWORD , "Longitudinal Studies") OR LIMIT-TO (EXACTKEYWORD , "Child Development") OR LIMIT-TO (EXACTKEYWORD , "Children") OR LIMIT-TO (EXACTKEYWORD , "Follow-Up Studies") OR LIMIT-TO (EXACTKEYWORD , "Motor Skills") OR LIMIT-TO (EXACTKEYWORD , "Motor Development") OR LIMIT-TO (EXACTKEYWORD , "Muscle Strength") OR LIMIT-TO (EXACTKEYWORD , "Childhood") OR LIMIT-TO (EXACTKEYWORD , "Child Health") OR LIMIT-TO (EXACTKEYWORD , "Fitness")) AND (LIMIT-TO (DOCTYPE , "ar"))*	17.902

	**Database**	**PsycINFO**	**Number of articles reached**
	**Descriptors/keywords**		
**1#**	**Any Field**: "physical fitness" OR strength OR motor OR "motor fitness" OR "cardiorespiratory fitness" OR flexibility OR muscular OR "muscular fitness" OR "musculoskeletal fitness" OR "muscle Strength" OR "muscular endurance" OR agility OR speed OR "physical performance" OR "health-related fitness"		158.097
	Filters> Stipulated time = last 10 years/ Apply equivalent subjects		
**2#**	**Any Field**: tracking OR stability OR longitudinal OR cohort OR "prospective studies"		176 348
	Filters> Stipulated time = last 10 years / Apply equivalent subjects		

**3#**	**Any Field**: child OR "school age" OR "school-age children" OR "primary school"	311 627
	Filters> Stipulated time = last 10 years/ Apply equivalent subjects	
**4#**	**1# AND 2# AND 3#**<br>*Stipulated time = last 10 years/ Apply equivalent subjects*	5.753

	**Database**	**Web of Science**	**Number of articles reached**
	**Descriptors/keywords**		
**1#**	**All fields=** "physical fitness" OR strength OR motor OR "motor fitness" OR "cardiorespiratory fitness" OR flexibility OR muscular OR "muscular fitness" OR "musculoskeletal fitness" OR "muscle strength" OR "muscular endurance" OR agility OR speed OR "physical performance" OR "health-related fitness")		1.402.011
**2#**	**All fields=** tracking OR stability OR longitudinal OR cohort OR "prospective studies")		1.397.284
**3#**	**All fields=** (Child OR "school age" OR "school-age children" OR "primary school")		1.233.187
**4#**	**#1 AND #2 AND #3**		11.708
	Stipulated time = last 10 years.		8.042

Total = 33.995

Supplementary file S3.

Methodological analysis and risk of bias of the included studies.

**Reference**	Item 1	Item 2	Item 3	Item 4	Item 5	Item 6	Item 7	Item 8	Item 9	Item 10	Item 11	Item 12	Item 13	Item 14	nº of Yes
Basterfield et al. (2022) [43]	Yes	Yes	Yes	Yes	No	Yes	Yes	Yes	Yes	Yes	Yes	No	No	Yes	11
Drenowatz et al. (2021) [44]	Yes	Yes	Yes	Yes	No	Yes	Yes	Yes	Yes	Yes	Yes	No	Yes	Yes	12
Hohmmann et al. (2021) [45]	Yes	Yes	NR	Yes	No	Yes	Yes	Yes	Yes	Yes	Yes	No	No	Yes	10
Jaakkola et al. (2021) [46]	Yes	Yes	Yes	Yes	Yes	Yes	Yes	Yes	Yes	Yes	Yes	No	No	Yes	12
Jarnig et al. (2022) [47]	Yes	Yes	Yes	Yes	No	Yes	Yes	Yes	Yes	Yes	Yes	No	Yes	No	11
Jarnig et al. (2022) [48]	Yes	Yes	Yes	Yes	No	Yes	Yes	Yes	Yes	Yes	Yes	No	Yes	Yes	12
Jarnig et al. (2022) [49]	Yes	Yes	Yes	NR	No	Yes	Yes	Yes	Yes	Yes	Yes	No	Yes	No	10
Reisberg et al. (2020) [50]	Yes	Yes	No	Yes	No	Yes	Yes	Yes	Yes	Yes	Yes	No	No	No	9
Reyes et al. (2023) [51]	Yes	Yes	Yes	Yes	No	Yes	Yes	Yes	Yes	Yes	Yes	No	Yes	Yes	12
Rodrigues et al. (2016) [52]	Yes	Yes	NR	Yes	No	Yes	Yes	Yes	Yes	Yes	Yes	No	Yes	Yes	11
Roth et al. (2018) [53]	Yes	Yes	Yes	Yes	No	Yes	Yes	Yes	Yes	Yes	Yes	No	No	No	10
Ruedl et al. (2018) [54]	Yes	Yes	Yes	Yes	No	Yes	Yes	Yes	Yes	Yes	Yes	No	No	Yes	11
Ruedl et al. (2019) [55]	Yes	Yes	Yes	Yes	No	Yes	Yes	Yes	Yes	Yes	Yes	No	No	Yes	11
Ruedl et al. (2022) [56]	Yes	Yes	Yes	Yes	No	Yes	Yes	Yes	Yes	Yes	Yes	No	No	Yes	11
Ruedl et al. (2023) [57]	Yes	Yes	Yes	Yes	No	Yes	Yes	Yes	Yes	Yes	Yes	No	Yes	Yes	12
Vanhelst et al. (2020) [58]	Yes	Yes	Yes	Yes	No	Yes	Yes	Yes	Yes	Yes	Yes	No	No	Yes	11
Werneck et al. (2019) [59]	Yes	Yes	Yes	Yes	Yes	Yes	Yes	Yes	Yes	Yes	Yes	No	Yes	Yes	13
Werneck et al. (2019) [60]	Yes	Yes	Yes	Yes	No	No	Yes	Yes	Yes	Yes	Yes	No	Yes	Yes	11
Total (%)	100%	100%	83.3%	94.4%	11.1%	94.4%	100%	100%	100%	100%	100%	0%	50%	78%	

NR, not reported.

Supplementary file S4.

Tracking Physical Fitness in children aged 5 to 12 years

Reference	Data Analysis	PF Tracking
Roth et al. (2018) [53]	Pearson autocorrelations	**Longitudinal cohort 1:**<br>**Boys:** 6 min run (0.470 to 0.499); 20 m sprint (0.552 to 0.633); standing long jump (0.592 to 0.635); push-ups (0.311 to 0.376); sit-ups (0.534 to 0.679); stand-and-reach (0.531 to 0.593).<br>**Girls:** 6 min run (0.339 to 0.517); 20 m sprint (0.340 to 0.494); standing long jump (0.392 to 0.624); stand-and-reach (0.616 to 0.655)<br>**Longitudinal cohort 2:**<br>**Boys**: 6 min run (0.625 to 0.565); 20m sprint (0.604 to 0.632); standing long jump (0.535 to 0.568); push-ups (0.297 to 0.331); sit-ups (0.480 to 0.523); stand-and-reach (0.512 to 0.576).<br>**Girls:** 6 min run (0.261 to 0.342); 20 m sprint (0.522 to 0.550); standing long jump (0.503 to 0.599); push-ups (0.235 to 0.356); sit-ups (0.411 to 0.414); stand-and-reach (0.615 to 0.624).
Vanhelst et al. (2020) [58]	The Wilcoxon signed-rank test	6 min (both sexes): P70 to P60, 5-s sprint P70 to P60 stability, standing long jump P70 to P60 (both sexes, steeper decline in girls), hopscotch Test P70 to P60 (both sexes).
Werneck et al. (2019) [59]	Kappa coefficient	PF: Boys (k = 0.444)<br>PF: Girls (k = 0.335)
Werneck et al. (2019) [60]	Kappa coefficient	Boys: 9 min run/walk (k = 0.357)<br>Girls: 9 min run/walk (k = 0.224)

PF = Physical fitness; r = Pearson auto-correlation coefficient; k = Cohen's kappa coefficient.
